# Impact of a cerebrospinal fluid diagnostic stewardship intervention on quantity of tests, length of stay, antibiotic prescriptions, and cost

**DOI:** 10.1017/ash.2025.17

**Published:** 2025-02-11

**Authors:** Aaron Pathak, Sabra Shay, Todd Lasco, Mayar Al Mohajer

**Affiliations:** 1 School of Medicine, Baylor College of Medicine, Houston, TX, USA; 2 Department of Clinical Intelligence, Premier Inc., Charlotte, NC, USA; 3 Department of Pathology & Immunology, Baylor College of Medicine, Houston, TX, USA; 4 Department of Medicine, Section of Infectious Diseases, Baylor College of Medicine, Houston, TX, USA

## Abstract

Redundant and low-value cerebrospinal fluid analysis for suspected meningitis can increase costs and antimicrobial use. Our diagnostic stewardship intervention limited available infectious disease cerebrospinal fluid assays to seven common tests, including a multiplex polymerase chain reaction panel. There was no significant difference in the cost of testing or clinical outcomes.

## Introduction

Cerebrospinal fluids (CSF) cultures are the gold standard for diagnosing meningitis and encephalitis but can take up to 48 hours to result.^1^ The rise of increasingly sensitive molecular assays with faster turnarounds has provided physicians with a plethora of tests for suspected meningitis and encephalitis with varying utility and cost.^2^ Excessive CSF testing for pathogens with low pre-test probability can lead to increased false-positive results, unnecessary antimicrobials, and excess healthcare spending (estimated median cost $383.50 per patient).^2–4^

Our institution made the BioFire® FilmArray® meningitis/encephalitis (FA/ME) panel (BioFire Diagnostics, LLC, Salt Lake City, UT) available in 2017, which detects 14 pathogens and was not restricted at our institution. Previously, we retrospectively evaluated cases (received CSF culture and FA/ME panel) and controls (received CSF culture alone) from 2017 to 2023.^5^ The FA/ME panel did not impact our institution’s antibiotic use or clinical outcomes but had a faster turnaround than traditional lab-based tests.^5^ The FA/ME panel was made available without active stewardship interventions; as such, many clinicians ordered redundant testing covered within the FA/ME panel, potentially raising hospital laboratory costs.^5^

To reduce unnecessary testing, we implemented a quality improvement intervention limiting available CSF infectious disease (ID) testing to seven common tests, including the FA/ME panel. This study aims to assess whether this intervention impacted the cost of CSF testing, length of stay (LOS), and empiric days of antibiotic therapy (eDOT) in patients with suspected meningitis/encephalitis.

## Methods

Our quasi-experimental study included patients from a quaternary academic medical center in Texas who received a lumbar puncture with subsequent ID testing. CSF testing was not restricted in the pre-intervention period (May 2023–October 2023). In the post-intervention phase (December 2023–February 2024), physicians were limited to ordering cell CSF count and differential, gram stain/bacterial culture, cryptococcal antigen, glucose, protein, FA/ME panel, and Venereal Disease Research Laboratory (VDRL) test if a serum Rapid Plasma Reagin (RPR) test was positive. Other CSF testing required ID consultation.

The primary outcome was the cost of ID CSF testing adjusted per patient. This was assigned using direct material costs to the hospital (Appendix A). Secondary outcomes included number of tests performed per patient, empiric days of antibiotic therapy (eDOT), and length of stay (LOS). eDOT was defined as the summation of days of each antibiotic prescribed for empiric treatment of suspected bacterial meningitis.

Mann-Whitney and Fisher’s exact tests were used to compare differences in patient baseline characteristics and test utilization pre- and post-intervention. Multiple regression models were fitted to assess the impact of the intervention on study outcomes (negative binomial for the number of tests per patient, linear regression for the squared root of cost per patient and log length of stay, and zero-inflated negative binomial for eDOT). Regression models controlled for demographics, admission location, and comorbidities. R version 4.3.3 (R Foundation for Statistical Computing, Vienna, Austria) was used for statistical analysis. This study was approved at our institution under Institutional Review Board Protocol H-51640.

## Results

A total of 245 patients were included (158 pre- and 85 post-intervention). There was no statistical difference between the two groups regarding demographics, admission location, or comorbidities (Table 1). Patients had a median age of 53.0, 54.0% were female, and 93.8% were admitted to the hospital.

**Table 1.** Baseline characteristics of patient population

	Pre-intervention (n = 158)	Post-intervention (n = 85)	*P*
Age (Years)- median (IQR)	52.15 (38.75–68.15)	54.70 (43.1–66.05)	0.543
Gender: Female- n (%)	83 (52.5)	48 (57.1)	0.502
Race- White- n (%)	104 (65.8)	54 (64.3)	0.886
Location			
Emergency Department- n (%)	12 (7.6)	3 (3.6)	0.301
Intensive Care Unit- n (%)	89 (56.3)	55 (65.5)	
Floor- n (%)	57 (36.1)	26 (31.0)	
Diabetes- n (%)	2 (2.4)	3 (1.9)	1
Transplant- n (%)	9 (5.7)	6 (7.1)	0.780
End Stage Renal Disease- n (%)	6 (3.8)	1 (1.2)	0.427
VP Shunt- n (%)	9 (5.7)	3 (3.6)	0.551
Encephalitis- n (%)	3 (1.9)	0 (0)	0.553
HIV- n (%)	12 (7.6)	4 (4.8)	0.588

After the intervention, the utilization of the FA/ME panel increased from 61.8% to 84.7% (*P* < .001). The median number of tests per patient pre- and post-intervention was 7 (*P* = .920). The range decreased from 1–42 before to 2–29 after the intervention. After controlling for confounders, there was no change in the number of tests per patient (IRR = .83, *P* = .063, Table 2), the squared root cost of tests per patient (B = 3.46, *P* = .068, Table 2), eDOT (IRR = .55, *P* = .102, Table 2), or the log LOS (B = .14, *P* = .461, Table 2).

**Table 2.** Multivariate predictors of the number of tests per patient, squared root cost of tests per patient, empiric days of therapy, and log-transformed length of stay

	Number of tests per patient (negative binomial)			Squared root- cost of tests per patient (linear)			Empiric DOT (zero-inflated model)			Log Length of Stay (linear)		
*Predictors*	*Incidence Rate Ratios*	*CI*	*P*	*Estimates*	*CI*	*P*	*Incidence Rate Ratios*	*CI*	*P*	*Estimates*	*CI*	*P*
(Intercept)	3.96	2.54 – 6.17	**<0.001**	16.11	7.25 – 24.97	**<0.001**	6.26	1.16 – 33.80	**0.033**	1.31	0.45 – 2.18	**0.003**
Study Period [Post Intervention]	0.83	0.69 – 1.01	0.063	3.46	–0.25 – 7.17	0.068	0.55	0.26 – 1.13	0.102	0.14	–0.23 – 0.50	0.461
Age (Years)	1.00	0.99 – 1.00	0.714	0.05	–0.06 – 0.16	0.359	0.99	0.97 – 1.01	0.467	0.00	–0.01 – 0.01	0.762
Gender [Male]	1.16	0.96 – 1.40	0.118	1.30	–2.36 – 4.97	0.485	0.67	0.33 – 1.36	0.263	0.38	0.03 – 0.74	**0.035**
Race [Others vs. White]	1.00	0.82 – 1.22	0.997	0.28	–3.52 – 4.08	0.885	0.80	0.38 – 1.67	0.556	0.04	–0.33 – 0.41	0.832
Location [Floor vs. ED]	1.01	0.70 – 1.47	0.943	7.37	–0.04 – 14.77	0.051	3.50	0.90 – 13.70	0.072	–0.18	–0.90 – 0.54	0.625
Location [ICU vs. ED]	0.89	0.60 – 1.32	0.552	7.01	–0.86 – 14.88	0.081	0.82	0.20 – 3.28	0.777	0.93	0.16 – 1.70	**0.018**
Diabetes	1.96	1.08 – 3.61	**0.028**	5.23	–8.25 – 18.70	0.445	0.42	0.04 – 3.99	0.449	–0.11	–1.42 – 1.20	0.869
Transplant	1.93	1.39 – 2.70	**<0.001**	9.86	2.63 – 17.09	**0.008**	0.79	0.21 – 2.90	0.720	0.79	0.08 – 1.49	**0.028**
ESRD	1.34	0.83 – 2.17	0.240	6.01	–4.26 – 16.28	0.250	0.73	0.11 – 4.73	0.745	0.35	–0.65 – 1.35	0.495
VP Shunt	0.62	0.38 – 0.99	**0.048**	–16.36	–24.67 – –8.05	**<0.001**	0.64	0.14 – 2.95	0.568	0.12	–0.69 – 0.93	0.773
Encephalitis	1.75	0.86 – 3.61	0.119	6.94	–8.52 – 22.40	0.377	0.10	0.01 – 1.48	0.094	–1.44	–2.95 – 0.07	0.061
HIV	1.62	1.17 – 2.24	**0.004**	12.05	4.96 – 19.14	**0.001**	0.26	0.08 – 0.86	**0.027**	–0.11	–0.80 – 0.58	0.757
Observations	231			231			231			231		
R^2^ Nagelkerke	0.275			0.185/0.140			0.619/0.596			0.211/0.168		

## Discussion

Limiting CSF tests available to physicians did not significantly affect costs or clinical outcomes in patients with suspected meningitis. Despite the reduced unapproved testing rate, the cost and number of performed tests were not statistically different due to increased utilization of the FA/ME panel in the post-intervention period. Since the FA/ME panel is done in-house, our intervention allowed physicians to receive CSF testing results faster without increasing total laboratory costs.

While our intervention did not show laboratory cost savings with increased FA/ME panel use, a meta-analysis showed potential cost savings with FA/ME use through metrics such as length of stay.^6^ Conversely, another study showed that increased FA/ME panel use led to increased direct testing costs but lowered antimicrobial costs, creating no net change in the total cost of care.^7^ Our intervention differed by increasing FA/ME panel utilization without increasing laboratory costs by requiring ID consult for other testing. Still, there were no associated changes in antibiotic prescriptions or LOS. The increase in FA/ME utilization mediated the lack of cost reduction at a cost of 854 USD at our institution, which was higher than previous literature at 214.44 USD (United States hospital) and 220 CAD (Canadian hospital).^7,8^ Future improvements to our intervention include panel limitations based on CSF white blood cell counts, which may safely decrease panel utilization without affecting clinical outcomes.^9,10^

Limitations to our study include being at a single center and our inability to account for seasonal variation over ten months. We were unable to assess whether the increase in FA/ME panel usage limiting cost savings was due to our intervention or increased clinician familiarity with the test. Clinicians were provided a list of approved assays including the FA/ME panel through an email detailing the intervention protocol, which could have prompted physicians to preferentially order all seven of the approved tests. Our institution’s cost of the FA/ME panel was higher than in previous literature, which could prevent cost reduction from our intervention.^6,7^

It is important to note that our intervention could create additional ID consults as required for CSF testing outside of the pre-approved diagnostics. Although we are unable to measure total consult numbers at our institution, this effect may be tempered by cases requiring nonstandard ID testing already necessitating ID physician consultation. Limited evidence also shows that the FA/ME panel could have lower sensitivities than individualized viral polymerase chain reaction (PCR) tests, which prevents fully removing all viral PCR tests that overlap with FA/ME panel coverage.^10^

Our study’s strengths include its large sample size compared to previous studies.^6,7^ We also employed multiple regression models to control for confounding variables. Finally, our quasi-experimental design with defined intervention periods strengthens our study by allowing us to assess the intervention’s impact in a real-world setting.

## Conclusion

Our intervention did not reduce the number of tests ordered or the cost of testing for patients with suspected meningitis. The decrease in unapproved tests was balanced by an increase in the FA/ME panel, preventing cost reduction. Future improvements should include diagnostic stewardship of the FA/ME panel which has a high direct cost to hospital systems.

## Supporting information

Pathak et al. supplementary material Pathak et al. supplementary material

## Supplementary material

For supplementary material accompanying this paper visit http://doi.org/10.1017/ash.2025.17. click here to view supplementary material
