# Supplementary material for: Impact of a cerebrospinal fluid diagnostic stewardship intervention on quantity of tests, length of stay, antibiotic prescriptions, and cost
Source: Antimicrob Steward Healthc Epidemiol. 2025 Feb 11;5(1):e35. doi: 10.1017/ash.2025.17 (PMC11822616; doi:10.1017/ash.2025.17)
Supplement: Pathak et al. supplementary material [file S2732494X25000178sup001.docx]

**Appendix A.** Direct cost of laboratory tests included in the intervention

| Test Name | Direct Laboratory Costs |
| --- | --- |
| **Tests Not Requiring ID Consultation** |  |
| CSF culture + gram stain | $49.63 |
| CSF cell count with differential | $35.00 |
| Cryptococcal antigen, CSF | $9.27 |
| Glucose, CSF | $3.10 |
| Protein, CSF | $3.00 |
| Meningitis/Encephalitis Panel | $854.00 |
| VDRL, CSF (Approved with Positive RPR) | $11.25 |
| RPR (Blood) | $7.84 |
| **Tests Requiring ID Consultation** |  |
| AFB culture + smear | $462.00 |
| Fungus culture + smear | $516.00 |
| Virus Culture | $34.35 |
| CMV PCR, quantitative | $61.25 |
| EBV Antibody, IgG | $8.98 |
| EBV Viral Load | $135.00 |
| Strep pneumoniae antigen | $125.58 |
| Fungal Panel | $63.98 |
| West Nile Virus, CSF, IgG and IgM | $19.60 |
| Varicella zoster PCR, qualitative | $100.00 |
| Varicella zoster antibody, IgM | $87.25 |
| Varicella zoster antibody, IgG | $87.25 |
| Treponema pallidum Antibody, IFA (CSF) | $190.91 |
| Lyme Disease Antibody (IgG), IFA (CSF) | $164.06 |
| HSV 1/2 PCR, Qualitative | $51.25 |
| Herpes simplex virus culture | $51.25 |
| Mycobacterium TB PCR Non-Respiratory | $251.30 |
| Fungitell R B-D-Glucan With Reflex to Titer | $82.98 |
| Cysticerosis antibody, IgG | $62.00 |
| St Louis Encephalitis Ab (IgG, IgM) | $692.10 |
| Enterovirus PCR, qualitative | $79.91 |
| RT-QuIC Prion | $641.87 |
| JC Virus Quantitative PCR | $250.00 |
| Cryptococcal Antigen Titer, CSF | $9.27 |
| Anaerobic culture | $2.09 |
| MVISTA Histoplasma Ag EIA | $15.62 |
| Toxoplasma Antibody IgG | $12.36 |
| Toxoplasma Antibody IgM | $18.60 |
| Aspergillus galactomannan antigen | $46.16 |
| Coccidioides Antibody (IgG), Immunodiffusion | $17.50 |
| HIV-1 PCR, Quantitative | $46.25 |
| Cytomegalovirus antibody, IgG | $10.00 |
| Herpesvirus 6 DNA, QN PCR | $112.26 |
